# Enhanced normalisation of CD4/CD8 ratio with early antiretroviral therapy in primary HIV infection

**DOI:** 10.7448/IAS.17.4.19480

**Published:** 2014-11-02

**Authors:** John Thornhill, Jamie Inshaw, Soonita Oomeer, Pontiano Kaleebu, David Cooper, Gita Ramjee, Mauro Schechter, Giuseppe Tambussi, Julie Fox, Jose Maria Miro, Jonathan Weber, Abdel Babiker, Kholoud Porter, Sarah Fidler

**Affiliations:** 1 Department of Medicine, Imperial College, London, UK; 2 MRC Clinical Trials Unit at UCL, Institute of Clinical Trials & Methodology, London, UK; 3 Medical Research Council, Uganda Virus Research Institute, Entebbe, Uganda; 4 Kirby Institute, University of New South Wales, Sydney, Australia; 5 HIV Prevention Unit, Medical Research Council, Durban, South Africa; 6 Hospital Escola Sao Francisco de Assis, Universidade Federal do Rio de Janeiro, Rio de Janeiro, Brazil; 7 Division of Infectious Diseases, Ospedale San Raffaele, Milan, Italy; 8 Guys and St Thomas’ NHS Trust, Kings College London, London, UK; 9 Hospital Clinic, University of Barcelona, Barcelona, Spain

## Abstract

**Introduction:**

Despite normalization of total CD4 counts, ongoing immune dysfunction is noted amongst those on antiretroviral therapy (ART). Low CD4/CD8 ratio is associated with a high risk of AIDS and non-AIDS events and may act as a marker of immune senescence [1]. This ratio is improved by ART although normalization is uncommon (~7%) [2]. The probability of normalization of CD4 count is improved with immediate ART initiation in primary HIV infection (PHI) [3]. We examined whether CD4/CD8 ratio similarly normalized in immediate vs. deferred ART at PHI.

**Material and Methods:**

Using data from the SPARTAC trial and the UK Register of HIV Seroconverters, we examined the effect of ART with time (continuous) from HIV seroconversion (SC) on CD4/CD8 ratio (≥1) adjusted for sex, risk group, ethnicity, enrolment from an African site and both CD4 count and age at ART initiation. We also examined that effect by dichotomizing HIV duration at ART initiation (ART started within six months of SC: early ART; ART initiated>six months after SC: deferred). We also considered time to CD4 count normalization (≥900 cells/mm^3^).

**Results:**

In total, 353 initiated ART with median (IQR) 97.9 (60.5, 384.5) days from estimated seroconversion; 253/353 early ART, 100 deferred ART. At one year after starting ART, 114/253 (45%) early ART had normalized CD4/8 ratio, compared with 11/99 (11%) in the deferred group, whilst 83/253 (33%) of early ART had normalized CD4 counts, compared with 3/99 (3%) in the deferred group. Individuals initiating within six months of PHI were significantly more likely to reach normal ratio than those initiating later (HR, 95% CI 2.96, 1.75 – 5.01, p<0.001). The longer after SC ART was initiated, the reduced likelihood of achieving normalization of CD4/CD8 ratio (HR 0.98, 95% CI 0.96 – 0.99 for each 30-day increase). CD4 count at ART initiation was also associated with normalization, as expected (HR 1.002, 95% CI 1.001 – 1.002, p<0.001). There was an association between normal CD4/CD8 ratio and being virally suppressed (<400 copies HIV RNA/ml) p<0.001. CD4 count normalization was also significantly more likely for those initiating early (HR 5.00, 95% CI 1.52 – 16.41, p=0.008).

**Conclusions:**

The likelihood of achieving normalization of CD4/CD8 ratios was increased if ART was initiated within six months of PHI. Higher CD4/CD8 ratio may reflect a more “normal” immune phenotype conferring enhanced prognosis and predict post-treatment control.
